# Acute Effect of a Single Dose of Tomato *Sofrito* on Plasmatic Inflammatory Biomarkers in Healthy Men

**DOI:** 10.3390/nu11040851

**Published:** 2019-04-15

**Authors:** Sara Hurtado-Barroso, Miriam Martínez-Huélamo, Jose Fernando Rinaldi de Alvarenga, Paola Quifer-Rada, Anna Vallverdú-Queralt, Silvia Pérez-Fernández, Rosa M. Lamuela-Raventós

**Affiliations:** 1 Department of Nutrition, Food Sciences and Gastronomy, School of Pharmacy and Food Sciences, University of Barcelona, 08028 Barcelona, Spain; sara.hurtado_17@ub.edu (S.H.-B.); mmartinezh8@gmail.com (M.M.-H.); zehfernando@gmail.com (J.F.R.d.A.); avallverdu@ub.edu (A.V.-Q.); 2 CIBER Physiopathology of Obesity and Nutrition (CIBEROBN), Institute of Health Carlos III, 28029 Madrid, Spain; 3 INSA-UB, Nutrition and Food Safety Research Institute, University of Barcelona, 08921 Santa Coloma de Gramenet, Spain; 4 Department of Endocrinology & Nutrition, CIBER of Diabetes and Associated Metabolic Diseases, Biomedical Research Institute Sant Pau, Hospital de la Santa Creu i Sant Pau, 08041 Barcelona, Spain; pquifer@santpau.cat; 5 Cardiovascular Epidemiology and Genetics Group, REGICOR, IMIM (Instituto Hospital del Mar de Investigaciones Biomédicas), 08003 Barcelona, Spain; sperez@imim.es; 6 Center for the Biomedical Research Network in Cardiovascular Diseases (CIBERCV), Institute of Health Carlos III, 28029 Madrid, Spain

**Keywords:** carotenoids, polyphenols, TNF-α, CRP, lycopene, β-carotene, bioavailability, Mediterranean, onion, extra virgin olive oil

## Abstract

*Sofrito* is a Mediterranean tomato-based sauce that typically also contains olive oil, onion, and garlic. The preparation of *sofrito* modifies the bioactive compounds (carotenoids and polyphenols) in the ingredients to more bioavailable forms, promoting *cis*-lycopene formation and polyphenol bioaccessibility. To evaluate the health benefits of this cooking technique, the effect of consuming an acute dose of *sofrito* on the inflammatory status was studied. In a clinical trial, 22 healthy male subjects consumed a single dose of *sofrito* (240 g/70 kg) after three days without ingesting any tomato products and following a low-antioxidant diet the day before the intervention. Plasma carotenoids and total polyphenol excretion (TPE) were evaluated, as well as the inflammatory biomarkers C-reactive protein (CRP), interleukin-6 (IL-6), interleukin 1β (IL-1β) and tumor necrosis factor-α (TNF-α). After the *sofrito* intake, a significant decrease in CRP (*p* = 0.010) and TNF-α (*p* = 0.011) was observed, but only TNF-α was inversely correlated with an increase in TPE and plasma *β*-carotene (not the major carotenoid, lycopene). The positive health effects of this tomato-based product may be attributed not only to lycopene, but to the bioactive compounds of all the ingredients.

## 1. Introduction

*Sofrito* is a sauce commonly used to prepare dishes in Mediterranean cuisine. It is based on tomato but also contains other ingredients, typically olive oil, onion, and garlic. The regular consumption of this sauce is included in a validated 14-item questionnaire to evaluate adherence to the Mediterranean diet [1,2].

Numerous studies have provided evidence for the protective role of tomato-based products and their bioactive compounds against the development of cardiovascular diseases and cancer [3,4], which is partly attributed to positive effects on inflammatory biomarkers [5,6,7].

The polyphenol and carotenoid profile of *sofrito* varies according to its composition [8], but as tomato is the principle ingredient, the major carotenoids are lycopene and β-carotene [8]. Carotenoids in food are mainly all-*trans* isomers, whereas *cis*-isomers predominate in the human organism [9]. Factors such as cooking practices and the food matrix promote the isomerization of carotenoids and their bioavailability [9,10,11], the latter being increased by the presence of lipids [12,13,14]. Our research group recently reported an enhanced formation of *cis*-lycopene in *sofrito* associated with the concentration of onion and the cooking time [15]. The total polyphenol content of tomatoes could be increased by processing [16], and the bioaccessibility of tomato polyphenols is enhanced by processing and oil addition [17,18,19]. Thus, due to its phytochemical content, *sofrito* seems to be a health-promoting component of the Mediterranean diet. 

Although the impact of tomato products on health has been mainly associated with lycopene [4,20,21], the other bioactive compounds present in *sofrito* could also be implicated [22,23]. Previous studies have reported inhibitory effects of dietary phytochemicals on nuclear factor kappa-light-chain-enhancer of activated B cells (NF-κB) activation in macrophages, resulting in a decrease in pro-inflammatory cytokines and chemokines like interleukin (IL)-6, IL-1β, and tumor necrosis factor (TNF-α) [24,25]. As these molecules stimulate the production of C-reactive protein (CRP) in the liver, which activates NF-κB [26], their reduction could diminish the CRP level. In the current work, it was hypothesized that phytochemical compounds present in *sofrito* could improve the baseline inflammation level. Thus, the aim of this study was to evaluate the effect of a single dose of 240 g/70 kg of *sofrito* on the regulation of inflammatory biomarkers in healthy humans and to identify the biomarkers responsible for these changes.

## 2. Materials and Methods

### 2.1. Sofrito Samples

*Sofrito* samples were supplied by Gallina Blanca (GB Foods, Spain) and consisted of a mix of tomato (50%), onion (37%), extra virgin olive oil (12%), and salt. Every sample was packed in a glass jar that contained 350 g of the *sofrito*. The nutritional and phytochemical composition of *sofrito* are provided in Tables S1 and S2.

### 2.2. Participants

The study was carried out with twenty-two healthy male volunteers aged between 18 and 32. All participants provided written informed consent in advance. Only men were enrolled in the trial to avoid effects related to hormonal fluctuations during the menstrual cycle [27,28,29]. Exclusion criteria were chronic illness or homeostatic disorder, history of cardiovascular diseases, hypertension or dyslipidemia, toxic habits (such as use of tobacco, alcohol, and drugs), and tomato or onion allergy or intolerance.

### 2.3. Intervention

In this open, uncontrolled and acute nutritional study, all participants ingested a single portion of *sofrito* (240 g/70 kg body weight) in a state of fasting. Before the intervention, volunteers avoided eating tomatoes and their by-products for three days. One day before the intervention, they also followed a low antioxidant diet, which was continued until the last blood sample was drawn (24 hours after consumption of *sofrito*). The details of the diet are presented in Supplementary Material Table S3. 

The study was run in the Department of Nutrition, Food Sciences and Gastronomy of the Food and Nutrition Torribera Campus, University of Barcelona (Spain), according to the principles of the Declaration of Helsinki. The protocol was approved by the Ethics Committee of Clinical Investigation of the University of Barcelona (Barcelona, Spain). The clinical trial was registered and given the International Standard Randomized Controlled Trial Number (http://www.isrctn.com/) of ISRCTN17867378.

### 2.4. Dietary and Physical Activity Assessments

In order to complete the dietary register, subjects were asked to fill out a three-day food recall on the day of the intervention. These were analyzed using PCN Pro software (Programa de Càlcul Nutricional Professional, Santa Coloma de Gramenet, Barcelona). Physical activity was measured by the validated Spanish version of the Minnesota Leisure-Time Physical Activity Questionnaire [30].

### 2.5. Extraction of Biological Samples

**Figure 1:**
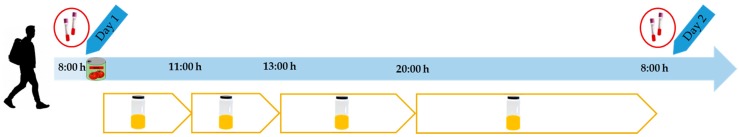
Timeline of sample collection before and after intake of *sofrito*. On the left, baseline extraction of blood and urine. On the right, sample drawn after *sofrito* consumption: collection of blood at 24 h and cumulative urine at 0–3, 3–5, 5–12, and 12–24 h.

Fasting blood and urine samples were drawn before *sofrito* consumption (baseline extraction) and up to 24 hours afterwards (Figure 1). Blood samples were collected via venipuncture in the arm through EDTA tubes, and plasma was separated after centrifugation at 1902 g for 15 min at 4 °C. Plasma and urine were aliquoted and stored at −80 °C until the day of analysis.

### 2.6. Clinical and Biochemical Evaluations

The diastolic and systolic blood pressure (DBP and SBP, respectively) and heart rate (HR) were measured in triplicate by a blood pressure monitor before and after the intake of a single dose of *sofrito*.

Biochemistry parameters in plasma were evaluated in an external laboratory (mdb.lab Durán Bellido, Barcelona). High density lipoprotein (HDL), low density lipoprotein (LDL), total cholesterol and triglycerides were analyzed by an enzymatic method. Urea and uric acid were analyzed by enzymatic and enzymatic/chromogen methods, respectively. Creatinine was determined by reaction kinetics of the Jaffe method (as modified by Larsen). Total proteins and albumin were measured by the endpoint biuret reaction and bromocresol green methods, respectively.

### 2.7. Analysis of Total Polyphenol Excretion in Urine

After a solid phase extraction (SPE) using a 96-well plate cartridge (Oasis MAX), total polyphenol excretion (TPE) analysis was performed in 2 mL of diluted (1:1) and acidified urine samples by the Folin–Ciocalteu method [31]. The spectrophotometry analysis was carried out using a Thermo Scientific Multiskan® Spectrum (Thermo Fisher Scientific, Vantaa, Finland, ref. 15019000) at a wavelength of 765 nm. The results are expressed as mg of gallic acid equivalent/L of urine (GAE/L). A cumulative urinary excretion curve for total polyphenols was calculated from 0 to 24 h. 

### 2.8. Quantitative Analysis of Carotenoids in Plasma

Carotenoids were extracted by liquid–liquid extraction from plasma samples collected at 0 h and 24 h [32]. Chromatographic analysis of carotenoids was performed by HPLC-UV-DAD, using an HP 1100 HPLC system (Hewlett-105 Packard, Waldbronn, DE) containing a quaternary pump coupled to a DAD G1315B. The separation was carried out with Milli-Q water, methanol (MeOH) and methyl-tert-butyl ether (MTBE) (Panreac Quimica S.A., Barcelona, Spain), according to a procedure previously validated in our group [32]. A Waters RP column YMC Carotenoid S-5 µm (250 mm × 4.6 mm) and a precolumn YMC Guard Cartridge Carotenoid S-5 µm (20 mm × 4.0 mαm) were used. 

Zeaxanthin (Extrasynthese, Genay, France), lutein, cryptoxanthin, *α*-carotene, *β*-carotene 9- and 13-*cis*-*β*-carotene (Sigma-Aldrich, St. Louis, MO, USA), lycopene (Fluka, Bucks, Switzerland), and 5-*cis*-lycopene (CaroteNature GmbH, Münsingen, Switzerland) were used as standards. These were pooled and prepared in synthetic human plasma (Sigma-Aldrich, St. Louis, MO, USA).

The sensitivity of each analyte was 0.703 µmol/L (lutein), 0.352 µmol/L (zeaxanthin), 0.362 µmol/L (cryptoxanthin), 0.480 µmol/L (*trans*-*β*-apo-8’-carotenal), 0.745 µmol/L (13-*cis*-*β*-carotene), 0.373 µmol/L (9-*cis*-*β*-carotene and *trans*-*β*-carotene), and 0.186 µmol/L (*trans* and *cis*-lycopenes) [32].

### 2.9. Determination of Plasmatic Inflammatory Biomarkers

Plasmatic C-reactive protein (CRP) was measured by an immunoturbidimetric method from external services (mdb.lab Durán Bellido) at baseline and 24 h after consumption of *sofrito*.

The concentrations of interleukin-6 (IL-6), tumor necrosis factor-*α* (TNF-*α*) and interleukin 1β (IL-1β) were assayed in plasma using the Immunoassay Kit (R&D Systems Inc., Minneapolis, USA, refs. HS600B, HSTA00E, HSLB00D). The sensitivity of each analyte was 0.110 pg/mL, 0.049 pg/mL, and 0.063 pg/mL, respectively. Plasma samples were assayed in duplicate. 

### 2.10. Statistical Analysis

Normality of distribution was assessed by a Shapiro–Wilk test. In order to compare baseline and post-intervention values, a linear regression analysis was used for the normal variables (SDP and DBP, HR, HDL, uric acid, albumin and TNF-α). Non-normal variables were assessed by a non-parametric Wilcoxon signed-rank test. The log-transformed TPE variable was analyzed by a Bonferroni post hoc test to compare the excretion of total polyphenols at the different time points. Statistical analyses were performed with SPSS Version 23.0 for Windows (SPSS Inc., Chicago, IL, USA). 

The correlation analysis was carried out using a correlation matrix that considers repeated measures [33] with software R, version 3.4.2. The Pearson coefficient (r) was calculated. Significant correlations (*p* < 0.05) are shown in the results.

## 3. Results

### 3.1. Characteristics of Participants

Table 1 provides anthropometric measurements of the participants, as well as the degree of adherence to the Mediterranean diet, level of physical activity and nutrient intake at the study baseline. 

### 3.2. Clinical Measures

Clinical measures are shown in Table 2. Systolic blood pressure did not change after consumption of *sofrito* compared to baseline, whereas diastolic blood pressure decreased (*p* = 0.006). The total cholesterol was lower after the intervention, mainly due to a reduction of HDL (*p* = 0.005 and 0.015), although significant changes in LDL and triglycerides were not observed. Urea, total proteins, and albumin decreased significantly (*p* = 0.023, 0.011, and 0.015).

### 3.3. Phenolic Excretion in Urine

**Figure 2:**
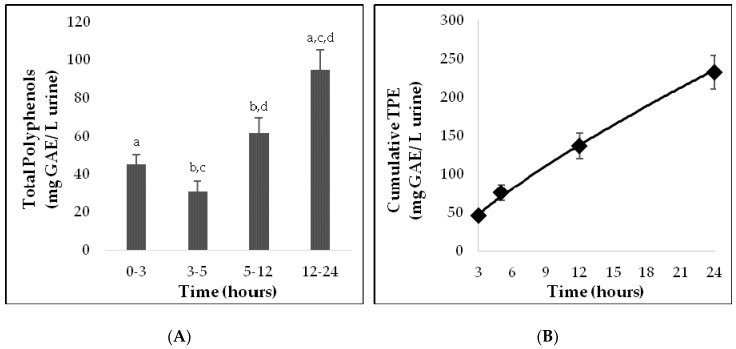
(**A**) Concentration of total polyphenols in the cumulative urine after consumption of a single serving of *sofrito*. (**B**) Cumulative urinary excretion curve of total polyphenols. Urinary total polyphenol excretion is shown: 0–3 (3 h), 0–5 (5 h), 0–12 (12 h), and 0–24 (24 h). The same letters (a–d) refer to statistically significant differences as follows. a: *p* = 0.001; b: *p* = 0.003; c: *p* <0.001, and d: *p* = 0.048. Data are expressed as mean + SEM.

As a biomarker of polyphenol intake from the *sofrito*, the urinary excretion of total polyphenols from baseline until 24 hours after consumption was analyzed (Figure 2A). The TPE increased from baseline to 5 h after intake, and even more so from 12 to 24 h (*p* = 0.001), as illustrated by the cumulative curve in Figure 2B.

### 3.4. Quantification of Carotenoids in Plasma

Levels of carotenoids in plasma were evaluated before and after *sofrito* consumption (Table 3). All target carotenoids increased after the intervention, the predominant ones being 5-*cis*-lycopene, *β*-carotene and *trans*-lycopene. Total lycopene increased from 43% to 59% of total carotenoids. Xanthophylls, lutein, and cryptoxanthin increased significantly (*p* = 0.001 and 0.012) and zeaxanthin was detected in plasma after *sofrito* consumption but not at baseline. In all participants, a high concentration of carotenes was found at 24 h of *sofrito* intake. Notably, the changes in *trans*-*β*-carotene were highly significant (*p* < 0.001), and the 9-*cis*-isoform and 13-*cis*-*β*-carotene were observed after the intervention but not at baseline. Other carotenes that increased were *trans*-lycopene (*p* < 0.001) and 13- and 5-*cis*-lycopene (*p* = 0.005 and <0.001). The concentration of 9-*cis*-lycopene was detected after the consumption of *sofrito* but not at baseline. In addition, the total *cis*-lycopene was significantly higher (*p* < 0.001), as was the total lycopene (*p* < 0.001). In summary, the increase in carotenoid concentration was statistically significant after the consumption of *sofrito*, total carotenoids being three-fold higher compared to the baseline level (*p* < 0.001).

### 3.5. Inflammatory Biomarkers

**Figure 3:**
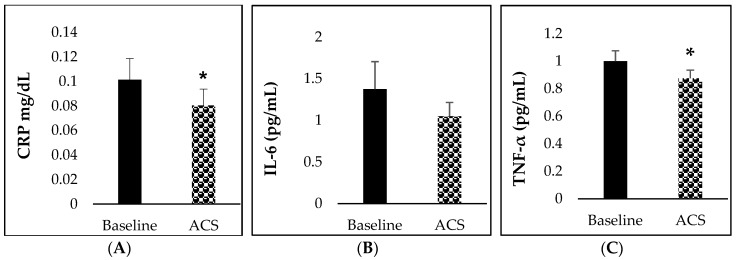
Concentration of inflammatory biomarkers at baseline and after consumption of *sofrito*. (**A**) CRP, (**B**) IL-6 and (**C**) TNF-α. Data are mean ± SEM, * *p*-value < 0.05. ACS: After consumption of *sofrito* (at 24 h). CRP: C-reactive protein; IL: interleukin; TNF-α: tumor necrosis factor alpha.

After the consumption of *sofrito*, all the inflammatory biomarkers studied decreased compared to the baseline. However, the changes were only significant for CRP (*p* = 0.010) and TNF-*α* (*p* = 0.011) (Figure 3A and 3C), but not IL-6 (Figure 3B). In no case was IL-1*β* detected (data not shown).

### 3.6. Correlations between Inflammatory Biomarkers and Bioactive Compounds

**Figure 4:**
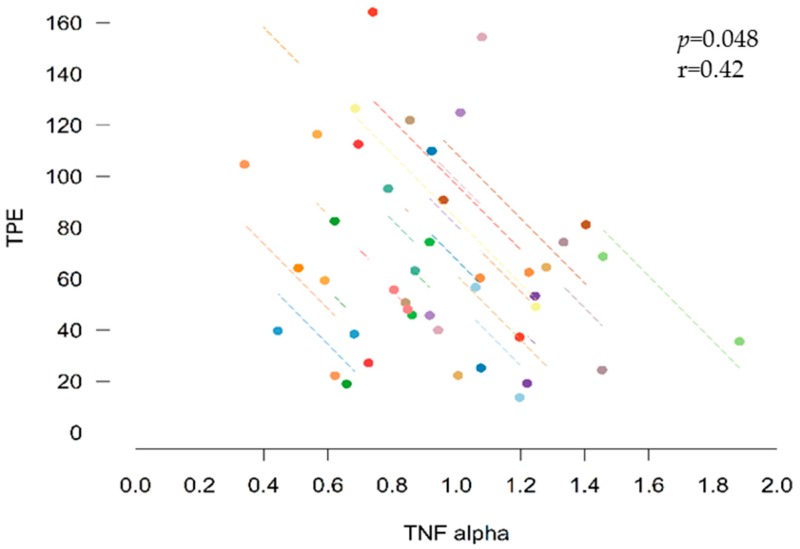
Correlation between tumor necrosis factor alpha (TNF-α) and total polyphenol excretion (TPE).

In order to evaluate the role of TPE in the decrease of inflammatory biomarkers, a correlation study was performed. The reduction of TNF-*α*, but not IL-6 or CRP, was inversely correlated with the excretion of total polyphenols. Figure 4 shows the negative correlation between TNF-*α* and urinary TPE (r = −0.42, *p* = 0.048).

**Figure 5:**
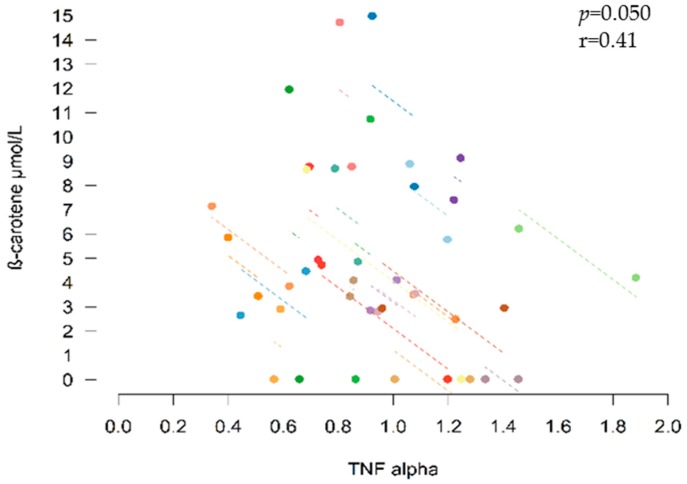
Correlation between TNF-*α* and *β*-carotene.

In addition, an inverse correlation was observed between the concentration of *β*-carotene in plasma and TNF-*α*, but not CRP and IL-6 (r = −0.41, *p* = 0.05) (Figure 5). 

## 4. Discussion

In this clinical trial, healthy individuals showed higher carotenoid levels in plasma 24 h after the consumption of *sofrito*. Arranz et al. reported higher plasma concentrations of *cis*-isomers of lycopene but not of other *cis*-carotenoids after the ingestion of tomato sauce without onion and garlic [12]. In the current study, both *cis*- and *trans*-isoforms of *β*-carotene and lycopene increased after *sofrito* intake. Not detected at baseline, 13 and 9-*cis*-isomer of *β*-carotene and 9-*cis*-lycopene were quantified after the intervention. 

TPE levels had increased significantly at 12-24 h after *sofrito* intake compared to the baseline. In a previous study by our group, also in healthy volunteers, changes in polyphenol excretion were noted earlier, at 6-12 h after the consumption of a tomato-based product without onion [19]. Thus, the presence of polyphenols from onion in the *sofrito* could have a delaying effect on the urinary TPE [34].

It was initially hypothesized that phytochemicals in *sofrito* could affect the regulation of systemic pro-inflammatory markers through the inhibition of NF-κB, triggering a decrease in cytokines, chemokines and CRP [24,25,26]. After the *sofrito* intake, a significant reduction of CRP and TNF-*α* biomarkers (*p* = 0.010 and 0.011) in plasma was observed, but not of IL-6. The decrease in TNF-*α* was inversely correlated with TPE and the level of *β*-carotene.

In a previous study, both CRP and TNF-*α* decreased significantly in healthy volunteers after the consumption of tomato juice twice daily for 2 weeks [5]. In obese and overweight participants who consumed tomato juice for 20 days, the concentration of TNF-*α* decreased, and in obese subjects the level of IL-6 was significantly reduced, but no changes in CRP were observed in either case [35]. In contrast, in a parallel study, a significant decrease in CRP was observed in women (but not men) suffering from heart failure who consumed tomato juice for a month [36]. Gender-related differences in CRP have been previously described [37,38]. In a crossover trial, tomato paste attenuated the increase in IL-6 after a high-fat meal in healthy volunteers [6]. However, Valderas-Martínez et al. reported that healthy subjects showed a significant reduction in IL-6 only after consuming tomato sauce containing olive oil (a single dose), but not raw tomato or tomato sauce without olive oil [7]. A study in schoolchildren found that plasma β-carotene levels were inversely related to IL-6 and CRP, particularly the former, but not to TNF-α [39]. In patients with cardiovascular diseases, β-carotene was significantly correlated with IL-6 [40] and CRP [22]. In contrast, other authors did not find changes in these inflammatory biomarkers after interventions with tomato products or a high-tomato diet [41,42,43,44,45,46].

In summary, an acute effect on inflammatory biomarkers was observed at 24 h after the administration of a single dose of 240 g/70 kg of *sofrito*. Improvements in inflammatory biomarkers after the ingestion of tomato products have been attributed mainly to lycopene. However, the results reported here indicate that when the product contains other ingredients, such as onion and virgin olive oil, different bioactive compounds such as polyphenols and carotenoids such as *β*-carotene may be responsible for the anti-inflammatory effects.

A strong point of this work is that relatively few acute studies have been carried out on the impact of diet on inflammatory biomarkers in healthy humans. The significant reduction in TNF-*α* and CRP levels after the ingestion of a single dose of tomato-based *sofrito*, rich in bioactive compounds, suggests this Mediterranean sauce may contribute positively to the regulation of the inflammatory status, even in individuals with optimal health.

The main limitation of the study is the lack of controls. Nevertheless, the analyses were carried out 24 h after the intervention in the same conditions to avoid changes due to circadian rhythms, and the volunteers continued a low antioxidant diet after the consumption of *sofrito*. 

## 5. Conclusions

The addition of *sofrito* to dishes and cuisine could contribute to the maintenance of health. An improvement in inflammatory biomarkers, particularly CRP and TNF-*α*, was observed after a single dose of *sofrito*, even though the subjects were healthy. Beneficial effects of *sofrito* intake could be partly attributed to the presence of bioactive compounds (polyphenols and carotenoids) from the ingredients. Although the health-protecting role of lycopene from tomato has been extensively studied, other carotenoids such as β-carotene (also found in large amounts in tomato by-products) seem to modulate the regulation of the inflammatory status.

## Figures and Tables

**Table 1:** Anthropometric parameters of subjects, physical activity in leisure time according to the Minnesota questionnaire, and the mean nutrient intake from a 3-day food recall.

Characteristics	
Age (years)	23.64 ± 0.86
BMI (kg/m^2^)	24.91 ± 0.79
WHR	0.84 ± 0.01
MedDiet adherence (score) ^1^	8.5 ± 0.4
Physical activity in leisure time (METs/d)	746 ± 71
Energy (kcal/day)	2393 ± 129
Total fats (g/day)	106 ± 9
Saturated fats (g/day)	32 ± 3
Monounsaturated (g/day)	47 ± 4
Polyunsaturated (g/day)	19 ± 2
Cholesterol (mg/day)	301 ± 30
Carbohydrate (g/day)	256 ± 15
Protein (g/day)	100 ± 6
Fiber (g/day)	30 ± 2

Data are mean ± standard error of the mean (SEM). BMI: body mass index, WHR: waist–hip ratio, MedDiet: Mediterranean diet, METs/d: metabolic equivalent of task per day. ^1^ The score categories of adherence to the MedDiet are high (≥10), moderate (6–9), and low (≤5).

**Table 2:** Clinical parameters of all participants in the study.

Measures	Baseline	ACS	*P*
DBP (mmHg) ^#^	76 ± 2	71 ± 2	0.006 *
SBP (mmHg) ^#^	123 ± 2	124 ± 2	0.550
HR (bpm) ^#^	66 ± 21	62 ± 2	0.061
Total cholesterol (mmol/L) ^#^	3.83 ± 0.12	3.71 ± 0.12	0.005 *
HDL (mmol/L)	1.37 ± 0.06	1.32 ± 0.06	0.015 *
LDL (mmol/L) ^#^	2.04 ± 0.11	1.98 ± 0.11	0.130
Triglycerides (mmol/L) ^#^	0.94 ± 0.08	0.89 ± 0.06	0.531
Urea (mmol/L)	5.56 ± 0.30	5.08 ± 0.28	0.023 *
Creatinine (µmol/L)	76 ± 2	74 ± 1	0.157
Uric acid (µmol/L) ^#^	319 ± 11	319 ± 10	1.000
Total proteins (g/L) ^#^	73 ± 0.6	71± 0.6	0.011 *
Albumin (g/L) ^#^	47 ± 0.5	46 ± 0.5	0.015 *

Data are mean ± SEM. ACS: after consumption of *sofrito* (at 24 h). DBP: diastolic blood pressure. SBP: systolic blood pressure. HR: heart rate. * *p*-value < 0.05. ^#^ Data analyzed by linear regression. The remaining data were analyzed by the Wilcoxon test.

**Table 3:** Concentration of carotenoids in plasma at baseline and 24 h after consumption of *sofrito*.

Analyte	Baseline	ACS	*p*
Lutein (µmol/L)	1.12 ± 0.02	1.69 ± 0.27	0.001 *
Zeaxanthin (µmol/L)	n.d.	0.65 ± 0.19	-
Cryptoxanthin (µmol/L)	1.08 ± 0.12	1.34 ± 0.15	0.012 *
*trans*-*β*-carotene (µmol/L)	3.12 ± 0.58	6.64 ± 0.88	<0.001 *
13-*cis*-*β*-carotene (µmol/L)	n.d.	0.75 ± 0.15	-
9-*cis*-*β*-carotene (µmol/L)	n.d.	0.95 ± 0.22	-
*trans*-lycopene (µmol/L)	2.15 ± 0.30	6.33 ± 1.53	<0.001 *
5-*cis*-lycopene (µmol/L)	1.87 ± 0.28	7.93 ± 2.73	<0.001 *
13-*cis*-lycopene (µmol/L)	0.21 ± 0.11	2.08 ± 0.78	0.005 *
9-*cis*-lycopene (µmol/L)	n.d.	0.90 ± 0.58	-
Total *cis*-*β*-carotene (µmol/L)	n.d.	1.92 ± 0.33	-
Total *β*-carotene	3.45 ± 0.67	8.56 ± 1.13	<0.001 *
Total cis-lycopene isomers (µmol/L)	2.09 ± 0.32	10.91 ± 4.00	<0.001 *
Total lycopene (µmol/L)	4.24 ± 0.59	17.23 ± 5.50	<0.001 *
Total carotenoids (µmol/L)	9.97 ± 0.96	29.25 ± 6.45	<0.001 *

Data are mean ± SEM. ACS: after consumption of sofrito (at 24 h). * Significant differences when *p*-value < 0.05. Data were analyzed by the Wilcoxon test. n.d.: not detectable.

## Supplementary Materials

The following are available online at https://www.mdpi.com/2072-6643/11/4/851/s1. Table S1: Nutritional value of *sofrito*; Table S2: Comparison of phytochemical composition of tomato and *sofrito* samples; Table S3: Recommendations to follow a low antioxidant diet.
