# Supplementary material for: Acute Effect of a Single Dose of Tomato Sofrito on Plasmatic Inflammatory Biomarkers in Healthy Men
Source: Nutrients. 2019 Apr 15;11(4):851. doi: 10.3390/nu11040851 (PMC6520770; doi:10.3390/nu11040851)
Supplement: Supplementary file 1 [file nutrients-11-00851-s001.zip › Suplementary/Table S2.pdf]

**Table S2.** Comparison of phytochemical composition of tomato and *sofrito* samples.

| Phytochemicals | Tomato (mg/240 g fresh weight) | Sofrito (mg/240g fresh weight) |
|---|---|---|
| **Total polyphenols (mg)** | 24.5 | 60.4 |
| **Total carotenoids (mg)** | 5.04 | 59.2 |

Values of polyphenols and carotenoids quantified in our group
Data in mg/ dose administered
Dose administered=

Sofrito is 240 g per 70 kg of body weight
