# Supplementary material for: Acute Effect of a Single Dose of Tomato Sofrito on Plasmatic Inflammatory Biomarkers in Healthy Men
Source: Nutrients. 2019 Apr 15;11(4):851. doi: 10.3390/nu11040851 (PMC6520770; doi:10.3390/nu11040851)
Supplement: Supplementary file 1 [file nutrients-11-00851-s001.zip › Suplementary/Tables S1.pdf]

Table S1. Nutritional value of *sofrito*.

	**Nutrients**
**Energy**[1]	278.4
**Total fats**[2]	22.56
**Saturated fats**[2]	3.6
**Carbohydrate**[2]	13.44
**Protein**[2]	3.36
**Fibre**[2]	3.84

Values of nutrients have been obtained from the product label
[1]Kcal/dose administered; [2]g/dose administered
Dose administered= 240 g per 70 kg of body weight
